# Study on Medical Students' Knowledge, Performance, and Understanding of Parasitic Diseases, With a Focus on *Leishmania*

**DOI:** 10.1155/japr/2660508

**Published:** 2025-10-07

**Authors:** Safa Najafi, Batool Ghafle Marammazi, Mohammad Hossein Feiz Haddad, Elham Farhadi, Sirous Rafiei Asl

**Affiliations:** ^1^Nursing Care Research Center in Chronic Diseases, Ahvaz Jundishapur University of Medical Sciences, Ahvaz, Iran; ^2^Department of Medical and Surgical Nursing, School of Nursing and Midwifery, Ahvaz Jundishapur University of Medical Sciences, Ahvaz, Iran; ^3^Student Research Committee, Ahvaz Jundishapur University of Medical Sciences, Ahvaz, Iran; ^4^Infectious and Tropical Diseases Research Center, Health Research Institute, Ahvaz Jundishapur University of Medical Sciences, Ahvaz, Iran; ^5^Department of Parasitology, School of Medicine, Ahvaz Jundishapur University of Medical Sciences, Ahvaz, Iran; ^6^Clinical Research Development Unit, Golestan Hospital, Ahvaz Jundishapur University of Medical Sciences, Ahvaz, Iran; ^7^Cancer, Environmental and Petroleum Pollutants Research Center, Ahvaz Jundishapur University of Medical Sciences, Ahvaz, Iran; ^8^Department of Pathology, School of Medicine, Ahvaz Jundishapur University of Medical Sciences, Ahvaz, Iran; ^9^Alimentary Tract Research Center, Clinical Sciences Research Institute, Ahvaz Jundishapur University of Medical Sciences, Ahvaz, Iran

**Keywords:** endemic diseases, *leishmaniasis*, parasitic diseases

## Abstract

**Introduction:**

*Leishmaniasis* is a prevalent parasitic disease in the tropical and subtropical regions of Iran and represents a major public health concern in the country. This study is aimed at assessing the level of knowledge, performance, and recognition among medical students regarding parasitic diseases, with particular emphasis on *Leishmania* infections.

**Methods:**

This descriptive-analytical study was conducted at Ahvaz University of Medical Sciences, focusing on a population of medical students, including interns and residents. Data were collected using a researcher-made questionnaire consisting of three main sections: demographic information (including age, gender, education level, history of student parasitic disease, student's previous clinical exposure to patients with parasitic infections, parasitology course grade, and main sources of information), knowledge, and performance assessment. Data were analyzed using SPSS software Version 26. Independent *t*-test, chi-square, and one-way ANOVA were used to assess the relationships between demographic variables and levels of awareness and performance. A *p* value of less than 0.05 was considered statistically significant.

**Findings:**

According to the results, 50.2% of the participants were female, most of whom were single, with a mean age of 25.52 ± 3.107 years. The average scores for knowledge and performance regarding parasitic diseases—with an emphasis on *Leishmania*—were 13.681 ± 3.706 and 16.195 ± 0.902, respectively. These findings indicate that the students possessed a moderate level of knowledge but demonstrated good performance. Notably, awareness levels were significantly higher among female students and interns.

**Conclusion:**

It is important to highlight that enhancing the awareness of medical students through face-to-face training, mobile-based education, and seminars can significantly reduce the risk of leishmaniasis transmission. By bridging the gap between public awareness and practical implementation, these future healthcare professionals who play a crucial role in education and treatment will be better qualified to control parasitic diseases.

## 1. Introduction

*Leishmaniasis* is a zoonotic parasitic disease transmitted to mammals, including humans, by protozoan parasites of the genus *Leishmania* and by sandfly vectors belonging to the genus *Phlebotomus* in the Old World [1–4]. According to the World Health Organization report in 2024, more than one million new cases of cutaneous leishmaniasis are reported worldwide annually, of which more than 4000 cases of cutaneous leishmaniasis were reported in Iran in 2023, indicating the ongoing public health challenge posed by this disease [5, 6].

*Leishmania major* is the primary causative agent of zoonotic cutaneous leishmaniasis (ZCL), with *Phlebotomus papatasi* as its main vector [7]. Despite considerable efforts to control the disease, ZCL remains endemic in many regions, partly due to gaps in public knowledge and preventive behaviors [8].

Knowledge, attitudes, and practices (KAP) regarding ZCL critically influence the success of control programs [9]. Studies have consistently shown that inadequate awareness and misconceptions about the disease's transmission, symptoms, and prevention contribute to ongoing transmission cycles [10, 11]. For instance, many at-risk populations lack sufficient understanding of vector biology or the role of reservoir hosts, limiting their adoption of protective measures [12].

Evaluations of community KAP enable identification of educational gaps and misbeliefs that hinder effective disease management. Targeted educational interventions, especially among high-risk groups and healthcare personnel, have been shown to improve preventive practices and early treatment seeking [13]. Furthermore, medical students, as future healthcare providers, play a pivotal role in disseminating accurate information and fostering community engagement, underscoring the importance of assessing their knowledge and attitudes toward ZCL [14, 15].

In addition to community-level factors, behavioral determinants such as personal protective measures, environmental management, and prompt healthcare utilization significantly affect disease outcomes. Therefore, comprehensive public health strategies must integrate KAP assessment findings to design culturally appropriate and effective educational programs [16].

Given the ongoing burden of ZCL and the absence of a fully effective vaccine, prevention through informed behavior remains paramount. This study is aimed at assessing the current levels of KAP among medical students regarding ZCL, providing essential data to tailor educational curricula and enhance future disease control efforts in endemic areas [9, 13].

In accordance with these needs, the present study was conducted to assess the levels of knowledge, practice, and perception among medical students concerning parasitic diseases, with particular emphasis on *leishmaniasis*.

## 2. Methods

This work is a descriptive-analytical study and was carried out at the Faculty of Medicine at Ahvaz University of Medical Sciences. The research population consisted of medical students, including both interns and specialists, while the sole exclusion criterion was unwillingness to participate in the study.

The researchers initiated the study after receiving the ethical approval code from Ahvaz University of Medical Sciences and obtaining the necessary permits. Prior to the completion of the questionnaires, the objectives of the study were clearly explained to the participants, and their informed consent was obtained. Participants were assured of the confidentiality and anonymity of their responses. They were also informed of their right to withdraw from the study at any time without any consequences.

Data were collected using a researcher-designed questionnaire comprising three main sections. The first section gathered demographic information, including age, gender, educational level, history of parasitic infection, prior hospitalization due to parasitic diseases, final grade in the parasitology course, and sources of information. This information was obtained through self-reported questionnaires based on previous medical history records. The second section assessed participants' level of awareness regarding parasitic diseases, and the third section evaluated their performance in relation to preventive and practical aspects.

The content validity of the questionnaire was confirmed by expert review conducted by faculty members from the Department of Parasitology. To assess its reliability, the questionnaire was administered twice to a sample of 15 medical students, with a 1-week interval between the two administrations. Correlation coefficients were calculated between the two sets of responses, and items with correlation coefficients below 0.80 were revised to improve clarity and consistency.

The questionnaire scoring system was structured as follows: For the awareness section, each correct answer was awarded one point, while incorrect answers or “I don't know” responses received zero points. The total possible score for awareness ranged from 0 to 20. Based on the obtained scores, awareness levels were categorized into three groups: poor (score < 10), moderate (score 10–15), and good (score > 15). For the performance section, a 5-point Likert scale was used for each item, with responses ranging from “never” to “always.” The performance score was calculated as the mean of the item scores, resulting in an overall score range between 14 and 19.

Data were analyzed using SPSS software Version 26. Descriptive statistics such as frequency, percentage, mean, and standard deviation were used to summarize demographic variables and questionnaire responses. Inferential statistical tests including independent *t*-test, chi-square test, and one-way ANOVA were employed to assess relationships between demographic variables and levels of awareness and performance. A *p* value of less than 0.05 was considered statistically significant.

## 3. Findings

According to the findings, out of the total participants, 100 students (49.8%) were male, and 101 students (50.2%) were female. The majority of the students were single, accounting for 169 individuals (84.1%), while 32 students (15.9%) were married. The mean age of the participants was 25.52 years with a standard deviation of ±3.107. Regarding performance, the students achieved an average performance score of 16.195 ± 0.902. The minimum recorded performance score was 14, and the maximum was 19 (Table 1).

To assess the level of knowledge regarding parasitic diseases—with a specific emphasis on *Leishmaniasis*—based on gender, the Mann–Whitney *U* test was employed. As shown in Table 2, the mean knowledge score among female students was 14.405, while that of male students was 12.950. The significance level of the test was determined to be 0.004 (*p* = 0.004), indicating a statistically significant difference in knowledge scores between male and female medical students. These results suggest that female students possess a significantly higher level of knowledge about parasitic diseases, particularly *Leishmaniasis*, compared to their male counterparts (Table 2).

To examine the relationship between medical students' knowledge and performance scores regarding parasitic diseases in relation to age, the Spearman correlation test was applied. As presented in Table 3, a significant and positive correlation was identified between the total performance score and students' knowledge (*p* < 0.001). However, no statistically significant correlation was found between age and performance (*p* = 0.274) or between age and knowledge (*p* = 0.956) (Table 3).

To assess the level of awareness regarding parasitic diseases, with a focus on *Leishmaniasis*, among medical students based on their year of university entry, the Kruskal–Wallis test was utilized. The results presented in Table 4 reveal a statistically significant difference in awareness scores across different entry years (*p* = 0.013). Notably, the highest average awareness level was observed among students who entered the university in 2015 (Table 4).

## 4. Discussion

Leishmaniasis control requires the active collaboration of researchers and practitioners across the animal, human, and environmental health sectors. Utilizing knowledge, process, practice (KPP) questionnaires among students and healthcare professionals in Iran is essential to identify knowledge gaps, practice deficiencies, and varying perceptions regarding the disease within these groups [9, 17, 18]. This study is aimed at evaluating medical students' knowledge, performance, and awareness of parasitic diseases, with particular emphasis on leishmaniasis.

In this study, 50.2% of participants were female, mostly single, with a mean age of 25.52 ± 3.11 years. The average knowledge and performance scores related to parasitic diseases, especially leishmaniasis, were 13.68 ± 3.71 out of 20 and 16.20 ± 0.90 out of 19, respectively, indicating moderate knowledge but relatively good practical performance among medical students.

These findings are consistent with previous research. For instance, Nadim et al. assessed healthcare staff in Iranian provinces and reported moderate baseline KPP levels, which improved significantly after training interventions [19]. Hosseini et al. evaluated health liaisons in Esfarayen County and observed similar moderate scores in knowledge and performance [20]. Likewise, Erfanyantaghvaei et al. found an average level of awareness about leishmaniasis treatment among general practitioners in Mashhad and Chenaran [21]. Given that physician awareness critically influences community education efforts, insufficient knowledge among healthcare providers may negatively impact disease control.

Gender analysis revealed that female medical students demonstrated significantly higher knowledge scores than their male counterparts. This aligns with studies from Ecuador's general population [22] and research by Saberi et al. among students in a hyperendemic area of Iran, where females scored higher in knowledge, attitudes, and awareness regarding leishmaniasis symptoms, vectors, and reservoirs [23].

A significant difference in knowledge was also observed based on the year of university entry, with students admitted in 2015—many of whom were interns during the study—exhibiting the highest awareness levels. This suggests that clinical exposure during internship enhances knowledge acquisition.

Moreover, a positive correlation between knowledge and performance scores (*r* = 0.289, *p* < 0.001) was found, indicating that greater knowledge is associated with better practical adherence to preventive measures. This relationship has been supported by Saberi et al. [23] and Alzolibani et al., who demonstrated significant links between awareness, attitudes, and practices related to cutaneous leishmaniasis among healthcare providers in Saudi Arabia [24].

Interestingly, no significant correlation was found between age and knowledge or performance, consistent with Aribodor et al., who reported similar findings in Nigerian medical students [25].

Overall, these results underscore the necessity of ongoing targeted educational interventions for medical students to enhance their knowledge and performance concerning parasitic diseases. Incorporating focused workshops, hands-on training, and updated educational resources within the curriculum may strengthen competencies and contribute substantially to disease prevention and control efforts.

This study relied on self-reported questionnaires, which may introduce response bias. Future research should consider longitudinal designs with larger sample sizes and objective measures to better evaluate knowledge retention and behavioral outcomes.

## 5. Conclusion

Based on the results of the present study, the level of awareness among students was found to be average, whereas their performance was rated as good. The awareness of medical students regarding parasitic diseases, with an emphasis on *leishmaniasis*, was higher than that of interns. Additionally, a significant and positive correlation was observed between the total performance score and students' awareness. It is noteworthy that enhancing the awareness of medical students through face-to-face education, mobile-based learning, and the organization of seminars can empower this group—who are at the forefront of public education and treatment—to reduce the risk of *leishmaniasis* transmission by narrowing the gap between public awareness and performance. It is important to highlight that enhancing awareness can reduce the risk of leishmaniasis transmission. By bridging the gap between public knowledge and practical application, future healthcare professionals will be better qualified to control parasitic diseases through education and treatment.

## Figures and Tables

**Table 1:** Performance levels of medical students regarding parasitic diseases, with a specific focus on *Leishmania* infection.

**Variable**	**S** **t** **a** **n** **d** **a** **r** **d** **deviation** ± **mean**	**Min value**	**Max value**
Medical students' performance on parasitic diseases with emphasis on *Leishmania* (average)	16.195 ± 0.902	14	19

**Table 2:** Knowledge scores of medical students regarding parasitic diseases with emphasis on *Leishmaniasis*, stratified by gender.

**Variable**	**Gender**		**p** **value**
	**Female**	**Male**	
	**M** **e** **a** **n** ± **SD**	**M** **e** **a** **n** ± **SD**	
Awareness	14.405 ± 3.433	12.95 ± 3.348	0.004

**Table 3:** Correlation between knowledge scores and performance of medical students regarding parasitic diseases.

	**Age**	**Performance (overall GPA)**	**Awareness**
	**Correlation coefficient (** **p** **value)**		
Age	1		
Performance (overall GPA)	0.078 (0.274)	1	
Awareness	0.004 (0.956)	0.289 (<0.001)^∗∗^	1

⁣^∗∗^Significant coefficients at an error level of less than 0.01.

**Table 4:** Comparison of medical students' knowledge of parasitic diseases with emphasis on *Leishmaniasis* by year of university entry.

**Year of entry**	**Mean awareness score**	**Standard deviation**
2000	13.00	0.00
2011	8.00	4.242
2013	9.00	0.00
2015	15.66	2.309
2016	13.80	3.608
2017	14.00	4.478
2018	14.72	2.418
2019	13.95	3.851
2020	11.42	2.651
2021	13.27	3.673

*Note:* ANOVA results: *F* = 20.990, *p* = 0.013.

## Data Availability

The data that support the findings of this study are available from the corresponding author upon reasonable request.
